# The Nexus Between Human Resource Management Practices and Service Recovery Performance in Takaful Insurance Industry in Pakistan: The Mediating Role of Employee Commitment

**DOI:** 10.3389/fpsyg.2021.752912

**Published:** 2022-03-14

**Authors:** Jie Mao, Saeed Siyal, Munawer Javed Ahmed, Riaz Ahmad, Chunlin Xin, Samina Qasim

**Affiliations:** ^1^Southwestern University of Finance and Economics, Chengdu, China; ^2^School of Economics and Management, Beijing University of Chemical Technology, Beijing, China; ^3^Department of Business Administration and Information Studies, Greenwich University, Karachi, Pakistan; ^4^Department of Business Administration, Iqra University, Karachi, Pakistan

**Keywords:** human resources management practices, service recovery performance, employee commitment, COVID-19, takaful industry, Pakistan

## Abstract

Service recovery performance (SRP) is very important for the takaful insurance industry for maintaining and attracting new clients, which in turn serves as a competitive advantage for the survival and continued future of the businesses. If the insurance sector could not maintain SRP, then the competitive advantage of the organizations could be decayed. Therefore, under the theoretical foundation of equity theory and resource-based theory, this research has investigated the link between human resources management practices (HRMP) (such as human capital, training, job description, teamwork, empowerment, and rewards) and SRP directly and indirectly through the employee commitment. By using a convenient sampling technique data was collected from the employees working in the Takaful industry in Pakistan to empirically test the proposed hypotheses and validate the findings. Using cross research design and quantitative research approach. The Structural Equation Modeling (SEM) had presented the positive relationship between HRM practices and SRP. On the other hand, employee commitment had also mediated this relationship. As employee commitment is significantly mediated among most of the HRMP, this aspect is therefore considered to be a big contribution of the study in the context of Pakistan. Based on these findings, the current study has several important implications the practitioners and readers.

## Introduction

In recent years, the insurance business in Islamic nations has evolved as Islamic Insurance (Takaful), notably the sale of takaful, has risen dramatically in Southeast Asian countries such as Malaysia and Indonesia. Takaful is known as a rapidly growing industry in Islamic emerging economies that provides Shariah-compliant services and is highly endorsed by Muslim academics worldwide. Insurance that complies with shariah standards and is recommended by Islamic laws addresses the true meaning of brotherhood by protecting people’s money from disasters and protecting their assets (Mihardjo et al., 2020). Compared to the traditional insurance sector, the Takaful industry in Indonesia and Malaysia, which have a majority Muslim population in the Southeast Asian area, has a limited market share and growth rate (Djafri and Noordin, 2017). Because of the great potential and vast Muslim population, the Takaful sector must focus immediately on acquiring the market and penetrating as an appealing alternative choice while giving superior possibilities to rivals.

Takaful laws were adopted in Pakistan under the SECP in 2005. Prior to this, the insurance industry was dominated by ultra-conservative insurance companies. Now, 10 Takaful companies (including 2 family, 3 general, and 5 window family Takaful firms) are functioning in the market with a portfolio of Shariah-compliant products. In Pakistan, there are now 5 life insurance firms and 27 general insurance companies functioning under the private Conventional Insurance market. For the 2019–2020 fiscal year, the total premiums written by conventional insurance are 349 billion, whereas the total premiums written by Takaful companies are 30 billion (2019–2020 Insurance Association of Pakistan Yearbook). Takaful is rapidly expanding as a Shariah-compliant alternative to traditional insurance, and its market share is predicted to increase to up to 50% of the whole sector share over the next 5 years. The GDP ratio of the total insurance sector business is written at 0.92 percent, with Islamic insurance accounting for 10% of the overall insurance business. In Pakistan, the contribution of takaful insurance has increased during the last decade.

Although insurance penetration remains negligible in Pakistan, the number of complaints filed by policyholders against insurance providers has increased dramatically in recent years. “In 2015, the percentage of cases filed was 120 percent more than the previous year.” “In 2016, the number of complaints received till May increased by 200 percent compared to the same time in 2015 and by 350 percent compared to 2014,” noted the Federal Insurance Ombudsman in its annual report for 2015–2016 (The Express Tribune, 2016). Furthermore, according to Business Recorder (2020), the Securities and Exchange Commission of Pakistan (SECP) has received a record number of complaints about insurance businesses regarding service defections that are not recovered by insurance service staff. Due to an overabundance of inadequate service recovery complaints, the SECP advised insurance policyholders to go direct to the SECP for the filing of their complaints or grievances over an insurance policy against insurance firms, agents, and bank representatives.

The aforementioned complaint statistics suggest that takaful insurance businesses are not only competing with traditional insurance but also struggling to provide high-quality services. Complaint statistics also show a service failure in terms of inadequate service recovery, resulting in organizations losing a steady stream of revenue from their consumers based on their lifetime usage value (Ahmad et al., 2015). As a result, customers are more likely to be unsatisfied with the product/service and thus to be less loyal to the organization (Javed Ahmad et al., 2018).

Furthermore, literature suggests that successful service recovery is heavily reliant on FLE performance, which is referred to as service recovery performance (SRP) (Piaralal et al., 2016). SRP refers to the steps performed by FLEs to successfully handle customer complaints and return consumers to a satisfied state (Ahmad et al., 2019b), which leads to perceptual, intentional, emotive, and behavioral outcomes such as customer repurchase intention (Piaralal et al., 2016). Hence, the customer service employees play a significant role in the SRP of a life insurance company, sustaining its reputation by building the confidence of customers of these companies. Moreover, HRMP and SRP have also been of considerable importance toward increasing the operational capacity of an organization by empowering employees and enabling them to take ideal decisions by working in teams. Hence, HRMP and SRP directly pertain to the employee commitment in all companies in general and insurance companies in particular (Mihardjo et al., 2020).

Moreover, the previous studies had mainly focused on three human resource management practices namely; human capital, training, and rewards (Mihardjo et al., 2020). During the COVID-19 pandemic, good human resources practices played an important role in the survival and recovery of performance. The countries which had better HR resources during the pandemic played an important role in enhancing their organizational performance (Piaralal et al., 2016). In this regard, the study is conducted in Pakistan to show the importance of these specific practices at the time of the COVID-19 pandemic in the takaful industry. Therefore, as per the researchers’ best knowledge, a comprehensive study along with six human resources (HRMP) practices such as human capital, training, rewards, empowerment, teamwork, and job description has not been assessed yet, especially in Pakistan. Consequently, the purpose of this research is to test the mediating effect of employee commitment on the relationship among human resource management practices and SRP pertaining to the takaful industry in Pakistan during the COVID-19 pandemic. The current study will assist the insurance business (takaful) in establishing human resource practices among its agents in order to improve their performance. It is apparent from empirical findings that there is little research examining the influence of agent performance on SRP (Piaralal et al., 2016). The literature is deficient in terms of information and knowledge on the influential variables identified as beneficial for improving the performance of frontline staff.

## Literature Review

### Service Recovery Performance

SRP refers to the degree of personal assessment of the service an employee delivers to customers. It is also defined as the effective ability of a service employee in resolving service failures and getting back customer satisfaction (Ahmad, 2019). SRP enables organizations to retain outstanding employees in order to accomplish organizational objectives of obtaining new clients as well as satisfying existing clients (Ahmad et al., 2019b). The insurance sector is heavily reliant on its agents; their success is extremely important to insurance corporations. The SRP provides help in the improvement of operations and the achievement of organizational goals such as acquiring new clients, satisfying existing customers, as well as building strong customer–employee connections. The employee capabilities, behavior, and the influence of employees on clients all have an impact on service recovery. Therefore, employees perform a critical part in service recovery toward managing the complaints of customers, since it is a key aspect in defining the reputation and service quality of the firm. SRP literature has been written in a variety of businesses, including the New Zealand banking sector; according to Turkey’s health and banking sectors, stronger recovery performance may be achieved by putting a better rewards system in place, assuring empowerment, improving teamwork, and making effective and swift decisions. As the market is continually changing, every firm, including the insurance industry, must evaluate the most recent advancements in systems and competitive foundations. Moreover, ASEAN free trade agreements have intensified competition. As a result, Malaysian insurance companies, as well as takaful service providers, must evaluate newly implemented market reforms; non-responsive behavior will result in a loss of business. The SRP is influenced by effective HR practices such as Employee Retention (ER) because incorrect, inappropriate, and insufficient HR practices would lead toward the failure of the takaful industry.

Human capital (HC), as well as Reward Strategy (RS) also affect Employee Commitment (EC), which in turn influences the SRP of takaful agents in Southeast Asia. Sonnentag and Frese (2012) distinguish SRP as the theoretical as well as practical behaviors of employees; first, numerous uncontrollable elements influence employee results, second, situational restrictions influence employee performance in the insurance sector, and third, opportunities play a critical part in measuring SRP. Moreover, the conduct of insurance company personnel is taken into account when regulating SRP habits (Piaralal et al., 2014). The SRP has been described as a complex construct by the researchers, with prior studies examining several sorts of recovery attempts such as refunds, regret, timespan, clarification, compensation, understanding as well as efforts. A few studies have been conducted in the finance industry (Chen and Sivakumar, 2021), tourism industry (Lv and Wu, 2021), and education sector (Ma and Bennett, 2021). According to Piaralal et al. (2016), there is a shortage of studies and literature about the SRP of insurance (takaful) agents as well as insufficient awareness about significant factors. Therefore, there is a pressing need to look into HR policies that have an impact on takaful agents’ SRP in the Southeast Asian nations like Indonesia as well as Malaysia, which have a significant number of Muslim inhabitants.

### Employee Commitment and Service Recovery Performance

Employee commitment (EC) has been extensively described in the literature by earlier researchers, who stated that EC helps in objective attainment and operational efficiency. Both of these have a positive effect on productivity (Meyer et al., 2013). Earlier, throughout the previous few decades, researchers have focused on psychological study (Huang et al., 2012). Belonging to a workplace and organization is also defined as an employee’s dedication to a firm or a department within it, as well as attachment to that organization or workplace (Meyer et al., 2013). According to the Constituent Model of the Organizational Commitment (OC), an employee urge to connect themselves to their place of work as well as organizations is necessary for EC. Employees’ desires are referred to as affective commitment, the need is known to be continuous commitment, the further obligation is to stay is known as normative commitment. A devoted employee, according to Meyer and Allen (1991), is one who stays with the firm through thick and thin, goes to work on a regular basis, always performs their full duties daily (plus possibly extra), defends corporate assets, and shares business objectives. The EC is distinct in place of an individual’s ability to become involved at work in order to attain goals and a high degree of performance in a rapidly changing environment. Establishing an association between employee goals and organizational values can also be used to measure organizational Commitment (Mowday et al., 1979; Djafri and Noordin, 2017).

Bielby and Bielby (1992) define EC as an attachment that is established and sustained through a person’s identification by a part, value, performance, or organization in place of as a source of identity that is seen to be central among alternatives. The OC model includes three basic components, these being affective commitment, normative commitment, and continuance commitment, established by Meyer and Allen (1991). These components remain distinct from one another and are grouped under organizational commitment. Each component of the commitment influences employee intentions to stay with an organization, but the thinking of each employee differs (Djafri and Noordin, 2017). Gupta et al. (2014) also explored the influence of spiritual aspects at work on EC, as well as many academics focused on the association between workplace spirituality and employee fulfillment as well as EC and performance (Djafri and Noordin, 2017). This study found that workplace spirituality influenced both affective as well as normative commitment with spirituality being less effective for long-term commitment. According to the study, when spirituality is present, attachment to one’s workplace and coworkers rises, which leads to more loyalty as well as dedication. Previously, some empirical investigations have showed that EC leads to higher outcomes and performance; it also reduces employee turnover intentions, unproductive behavior, and absenteeism (Djafri and Noordin, 2017).

Additional effects of organizational commitment, according to several studies, include employee citizenship behavior, as well as their wellness and health (Becker et al., 2012). Since the examination of Cooper-Hakim and Viswesvaran (2005) experimentally evaluated as well as discovered a positive and significant relationship among these dimensions, scholars have found a link among organizational commitment as well as performance in empirical investigations. On the other hand, a positive association between performance and effective commitment has been examined empirically in research of Van Dick et al. (2009). Zayas-Ortiz et al. (2015) investigated the association between employee conduct and commitment among banking sector employees. The study discovered a significant and positive association between EC and civic virtue. The strongest association was found between citizenship behavior and civic virtue. In summary, several research studies on commitment and its consequences on performance have been undertaken in many industries and nations, with conclusions that have been established to remain unsatisfying, as indicated in the previous section of the present study. In this study, two Islamic countries, Malaysia and Indonesia, have been discussed in the “Southeast Asian context.” The relationship between EC and performance in the takaful business has been studied by a number of scholars, together with (Remli and Rosman, 2018). The studies looked at several components in the Southeast Asian takaful sector, such as innovation, market orientation, and employee personality factors, in order to determine and assess the performance of takaful agents and operators.

### Human Resource Management and Service Recovery Performance in the Takaful Industry

Globalization has exploded in popularity in current years, putting significant hurdles in front of every organization and exposing them to more competition. The “Human Resource Management” (HRM) performs a significant part in the operational as well as strategic activities of businesses in order to improve and maintain employee performance. Past research has paid more attention to the role of HR practices in improving OP in the service or manufacturing fields in a fast-changing environment. Additionally, researchers looked at the HRM practices as well as performance-related results in various countries and firms and found an association between HR practices and performance. There is still a discussion over the impact of numerous diverse techniques in any business (production or services) on employees and OP (Abu Keir, 2016). Earlier, numerous researchers have recommended inspecting HR practices associated with service industry performance for the purpose of better understanding the impact of the insurance industry associated HR practices and their influences at SRP for takaful organizations (Abu Keir, 2016; Djafri and Noordin, 2017; Remli and Rosman, 2018). The basic purpose of the current research is to examine service industry HR practices in order to determine the takaful industry’s SRP in Southeast Asia. Human capital, reward management, and employee retention are examples of HR practices, with employee commitment serving as a mediating factor.

### Human Capital, Employee Commitment, and Performance

Generally, people remain pressed to expend their energies mentally rather than in physical pursuits as a result of technological breakthroughs and digital emergence. Human capital refers to new and updated knowledge as well as information that does not appear in financial data but has a major impact on OP when compared to physical assets (Kalkan et al., 2014; Abbas et al., 2018). The contribution of human capital (HC) to the success of a firm can be analyzed to maintain good performance (Abbas et al., 2018; Mihardjo et al., 2020). According to Hashim et al. (2018) Human Capital (HC) is employees’ knowledge and skills for adding value to attain desired performance inside of any sector. Moreover, knowledge management in organizations is also a part of HC management (Abbas et al., 2018). The literature focused on the research at HC as well as its characteristics to promote competitiveness to increase employee performance to achieve objectives (Abbas et al., 2018). Studies on the association between management and HC abound in the literature (Ramadan et al., 2017). Knowledge capability has been defined in various studies as the ability of a firm to manage Rajesh et al. (2011) and Roos (2017) mentioned that HC is critical for EP to maintain a competitive advantage in a volatile and fast fluctuating organizational environment. The academics clarified HC in three basic categories such as human, structural, and customer capital. Bontis et al. (2000) and Hashim et al. (2018) contained spiritual capital, social and technological variables, whereas HC was defined as employees’ skills and expertise. The customer capital was defined as customer and supplier relationships, besides structural capital defined as an organization’s processes, approaches, values, as well as a whole working system. Abbas et al. (2018) stated that for determining the performance of any organization, the focus should be on the human element and consumers, along with social, technological, structural, and spiritual capital. Previous literature made evident that HC as the component of HRM has the capacity to influence the performance related behaviors of an individual. For instance (Mihardjo et al., 2020) claims that HRM practices, particularly HC is the significant predictor of SRP in the service sector. Therefore, in context of this study HC is operationalized as the predictor of SRP which might have influence on the SRP, and it is expected that HC will positively contribute to enhancing the SRP of service employees.

### Training, Employee Commitment, and Performance

Employee training is a planned organizational effort to enable employees to acquire certain competencies regarding their job, such as skills, knowledge, and behaviors that are essential toward effective job performance (Piaralal et al., 2016). Employees must improve their job-related skills or habits in order to obtain and build capabilities to provide ideal products or services to customers. Employee training to improve job-related talents and behavioral skills was shown to be critical in a worldwide corporate environment for acquiring a competitive advantage (Yavas et al., 2010). As stated by CheRusuli et al. (2018) Human Resource Development (HRD) practices of employees for the purpose of enhancing performance services because of competencies between hotel business frontline employees, existing research endows significant consequences in the association between practices of HRD and service performance by employees skills serving in place of as a mediating factor. According to the presented SRP study, takaful agents training for enhancing their performance has been acknowledged as critical and essential in the highly competitive atmosphere. Until that time, different scholars had conducted various studies on the association of multiple service organizations to address the significances of employee training as a factor in improving performance such as in the banking business (Al-Ababneh et al., 2018; Mihardjo et al., 2020). A positive association was observed among workplace connection and training for attaining EC (Hodgkinson et al., 2018; Messersmith et al., 2018). The academics have recommended looking at the employees training in service organizations as an example of employee professional development in order to improve employee effectiveness and reduce attrition (Mihardjo et al., 2020). Employee training permits them to improve their capabilities, knowledge, and skills and enhance their satisfaction level in their workplace; retention, as well as loyalty, could be attained successfully in workers in health care, logging organizations, and the tourism business (Ananthakrishnan et al., 2018; Sharma and Gursoy, 2018). In addition, researchers also acknowledge the value of training content in improving staff productivity by changing attitudes as well as behaviors. An organization must check the contents of training and also influences of training on employees’ effort, turnover intentions, and commitment (Bhatti et al., 2017).

### Reward Management, Employee Commitment, and Service Recovery Performance

Rewards defined as compensation, appreciation, prestige, and identity in society are all terms used to describe the money received in exchange for services provided by employees to businesses (Yavas et al., 2003). In order to achieve better performance, the rewards supplied by the company in exchange for employee efforts, for example, recognition, promotion advancement prospects, salary, and personal development should be able to match the requirements or expectations of workers (Piaralal et al., 2016; Ahmad, 2019). Employees’ decisions at work are heavily influenced by the money they receive in the exchange for their services. Effective rewards, as well as remuneration, enable organizations to acquire a competitive advantage through retaining competent staff (Bulimo, 2014; Ahmad and Zakaria, 2018; Njenga, 2018). Babakus et al. (2003) discovered compensation as a key aspect in delivering service toward consumers with quality as well as providing help to the employees in the workplace to perform in a better way. For the purpose of better service, reward plans were studied in education, banking, health, and services of life insurance on front line employees, as well as a direct impact on the SRP being observed (Piaralal et al., 2016; Bhatti et al., 2017; Njenga, 2018). The compensation was found as a substantial and positive impacting variable for motivation as well as job completion both in public and private sector organizations in Malaysia (Ogbari et al., 2018).

Researchers also urge that incentive management and employee objectives be aligned for the purpose of employee satisfaction (Samsudin and Ahmad, 2018). The current research studies the multiple variables that consist of promotion, benefits, salary, and appreciation toward encouraging employees of the Pakistani Commercial Banks (Karam, 2017). In the service business, rewards remain to be particularly significant for motivating personnel and complaints resolving (Piaralal et al., 2014). The research was conducted in the banking industry of several nations including Turkey and New Zealand; reward practices remain found to be a positive and significant association toward service advantage at the frontline workers at SRP in the service industry of Malaysia (Piaralal et al., 2016). According to the findings of Baron et al. (2005), New Zealand hospital nursing staff establish that rewards have a positive effect on SRP. In the same way, Piaralal et al. (2016) found rewards as a significant element in the life insurance sector of Malaysia. The rewards perform a substantial part in the emotional happiness as well as strongly influence service quality. It implies that the employees who are in demand and earn a high salary are deemed to be content and give outstanding service, which has a positive impact on SRP. In New Zealand, Ashill et al. (2005) discovered that rewarding FLEs had a beneficial impact on their SRP. According to Forrester (2000), monetary incentives have a substantial impact on the performance of life insurance company employees. It was found by Khalid et al. (2012) that pay had a strong correlation with job satisfaction and motivation in the water utility businesses, both public and private. According to Singh (2004), remuneration has a major impact on employee performance and suggested that companies pay attention to it in order to succeed and develop rapidly in the market. Since the explanation above suggests that rewards have a beneficial impact on service employees’ SPR, it is presumed that this is the case.

### Teamwork, Employee Commitment, and Service Recovery Performance

Teamwork has been defined as the litigations taken by a company for increasing the cooperation between the employees so that they can work together and exert their best efforts for the attainment of a common goal (Adi, 2018), the goal of the company. It has been asserted that teamwork has played a significant role in organizational performance, leading to the achievement of anticipated revenues and objectives of a company. Teamwork enables the employees in a company to identify organizational problems and to look for their solutions. Moreover, this cooperation and synergy between the employees, connect them to a greater degree, enabling them to share ideas with each other, ultimately leading to increased innovation and organizational productivity. This significance is evident from research performed on approximately 600 business students; some of them were asked to study in teams and share ideas, while others studied individually. Later it was found that the students who studied in teams had far better results in final exams as compared to students who studied individually.

Furthermore, teamwork has also been of substantial significance as it has a great impact on the staff involved in organizational problems, and ultimately results in better solutions to solve these problems, all leading to organizational success and increased SRP (Gera, 2011). This significance of teamwork was proved by research performed on a Malaysian life insurance company, which demonstrated that teamwork has a positive effect on SRP, and thus led to the increased attainment of the objectives of the life insurance company. Hence, the research implied that employee commitment in a life insurance company is highly increased by teamwork, hence leading to organizational success (Ahmad et al., 2019a). Additionally (Robbins and Judge, 2017) established that teamwork is defined as a group of individuals whose collective efforts result in higher performance than the sum of their individual inputs. In conclusion, cooperation results in superior performance than solo performance inside an organization or business.

### Empowerment, Employee Commitment, and Service Recovery Performance

Empowerment is referred to as giving power and resilience to the employees, giving them motivation to apply their experiences and instincts and freedom to take certain decisions, all leading to increased employee engagement, and hence organizational success (Ahmad, 2019). Employee empowerment is directly related to one of these variables, organizational success, employee commitment, and organizational commitment. An organization that empowers its employees is more likely to achieve success and to achieve their business objectives. Increased employee empowerment leads to increased confidence, participation, and performance from employees, enabling them to use their skills and experiences to solve organizational problems and hence aid organizational success. In order to evaluate the importance of employee empowerment, research was conducted on the 290 customer service employees of a Chinese life insurance company, which showed that employee empowerment was directly related to organizational commitment and organizational success, as the employees contributed more because the organization gave them the confidence to express their skills and the freedom to take necessary actions in case of organizational crises (Ahmad, 2019).

Moreover, another study was performed in nearly 300 city and country clubs, which demonstrated that increased employee empowerment was directly related to the performance of the employees, especially the customer service employees (Elziny, 2021). The research showed that the participation of employees greatly increased after the policy of employee empowerment was implemented in these city and country clubs, leading to higher employee commitment by employees and higher operational capacity. Moreover, it seemed that this new policy of employee empowerment gave employees a different sort of confidence to make certain decisions to deal with organizational problems. Hence, employee empowerment increased the productivity of the employees and increased organizational success (Boshoff and Allen, 2000).

Therefore, it has been asserted by researchers that the employee commitment in service companies such as life insurance companies has been decreasing, hence employee empowerment is necessary for the organizational success and SRP of these companies. Thus, empowering employees in a life insurance company can be of substantial importance, as it is a strategic tool to increase the SRP of the company. Increased empowerment enables the employees to use their skills to the maximum level, and the freedom to take necessary marketing actions to captivate the customers and to tackle other organizational problems. This not only leads to organizational success but also gives the managers fewer problems to deal with so that they can focus on the attainment of the objectives and anticipated revenues of the company. Hence, the research proved that employee empowerment can greatly increase the employee commitment of employees, all implicitly leading to increased SRP and increased operational capacity of an organization (Bhatti and Haider, 2014). Employee empowerment is an important element in organizations. It is advantageous to the organization since empowered employees will experience a sense of belonging, enthusiasm, and pleasure and employ their greatest innovations and thoughts (Ahmad et al., 2021). Furthermore, employee empowerment increases organizational commitment, task fulfillment, job participation, and work satisfaction (Ahmad et al., 2018).

### Job Description, Employee Commitment, and Service Recovery Performance

Within an organization, employees differ with respect to their job description because of their capabilities, skills, education, and experiences, and each of the employees in an organization based on the above characteristics differ with respect to their rank in the organization, and the compensation they receive. According to Ilany et al. (2020), a job description is an overview of the work’s information and the needs for its implementation by an employee as a result of the analysis, which includes the basic task of the job. Ilany et al. (2020) postulated that a job description may also be described as the outcome of job analysis, which is the act of gathering and processing information about a job in a written manner. Pennell (2010) defines job description as “the act of describing the work, the responsibility, and the nature of the employment field.” The tasks must be clearly defined in terms of their nature and the responsibilities that should be assumed by the employee performing the job, for employees to avoid making errors due to a lack of understanding of the work that they should be performing. According to the experts’ definitions of work descriptions above, it can be inferred that a job description is a piece of information about a job that includes details about the duties and obligations of the position.

Additionally, the researchers asserted that if a job is not explained properly, it may have an effect on job discontent, absence, poor participation, stress, turnover intentions, and SRP (Ashill et al., 2009). However, job descriptions may not always have a beneficial effect, but they do have a high degree of usefulness in enhancing employee commitment and SRP (Kumar et al., 2012; Ahmad, 2019). In a service context, a clear job description appears to be a good predictor of employee performance, since it has an indirect effect on the customer’s views of service delivery (Boshoff and Allen, 2000).

Empirical evidence suggests overwhelmingly that if a job description is not clearly defined, this will lead employees toward stress which ultimately affects the SRP of front line employees (Ahmad et al., 2019b). Alexander (2011) stressed that the level of an individual’s stress is increased when their job description is ambiguous and the employee is confused by receiving different sets of directions, and cannot ignore or violate one set of directions to follow another. Moreover, employees who have direct interactions with customers usually experience contradictory feelings during the fulfillment of job requirements (Piaralal et al., 2016; Ahmad, 2019). Such types of vague job descriptions may cause negative customer interactions with consequences for their satisfaction and loyalty (Piaralal et al., 2016). Hence, it is inferred from the above discussion that employees who have a clear job description can play a vital role in an organization, and a clear job description is essential for the organizational success and SRP as based on their employee commitment and commitment to the companies in which they are employed, especially the life insurance companies.

### Mediating Impact of Employee Commitment Among Human Capital, Training, Teamwork, Empowerment, Job Description, Rewards, and Service Recovery Performance

The present study consists of three key human resources practices; Human resources, training, and reward management in the Southeast Asia takaful industry that affects employee involvement and performance. Detailed relationships between independent variables (Human Capital, Training, and Rewards) and dependent variable RPS will be discussed further throughout the paper with the mediating role of employee commitment in the South Asian Countries’ industry.

In the past, researchers conducted large-scale research which is important for building efficiency and productivity (Jehanzeb and Mohanty, 2018; Li et al., 2018). For two decades, researchers were extensively interested to demonstrate the background and outcomes of employee participation through organizational psychological research (Huang et al., 2012; Owen et al., 2018; Hansen and Pihl-Thingvad, 2019). Employee commitment to the job is known as employee membership, and employers’ commitment feelings are described in the model for employee commitment as the desire for choice and duty. Employees’ relationship with their business (Meyer and Allen, 1991). Meyer and Allen (1991) defines their employee engagement, as those employees who remain with the organization, work regularly, protect company assets, share company objectives, and etc. It is clearly an advantage from a corporate perspective to have a dedicated workforce.

This study will discuss the mediating role of employee commitment in the takaful industry in Southeast Asia between main human resources practices and SRPs. Employee commitment provides benefits to companies because they have affected public sector workers, including healthcare, banking, and insurance, in the SRP (Piaralal et al., 2016). The direct connection between the staff commitment and SRP by public and private frontier workers was explored in various countries including New Zealand, Turkey, Pakistan, and Malaysia. A few studies have shown, however, that the SRP has little to do with obligations (Piaralal et al., 2016). Some studies have been conducted related to COVID-19 (Chen et al., 2021; Li et al., 2021). Predictors, including HR practices such as rewards, training, empowerment, and teamwork, have an effect on employee engagement, however. Employee engagement plays an important role, in particular, to improve customer service recovery, in contributing results related to performance.

### The Research Framework

**FIGURE 1:**
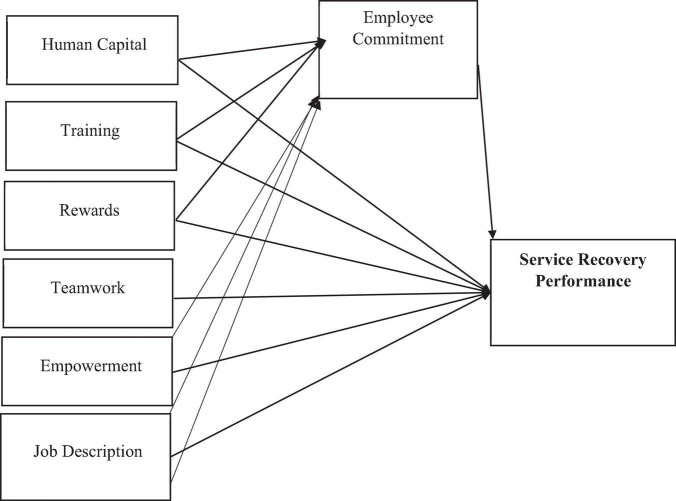
Research framework.

In the current study, the foundation of the theoretical framework has been taken based on the equity theory. This theory had been used for examining the services and satisfaction features due to failure and criticism and performance (Akhgari et al., 2018). Equity theory is based on equity exchange and recognition of performance results and contribution to the success of companies as individuals (Bulimo, 2014). In the present study, SRP has been hypothesized to increase employee engagement exchange. The second assumption is that SRP is improved by employee workplace commitment. The RBV Theory shows direct implications for human resources practices that influence the qualifications, attitudes, and behavior of outcomes. Resource-based approach theory argues that different resources influence the competitive edge based on expertise and skills. Therefore, focus on specific outcomes while applying significant human resource management practices, such as human capital, training, rewards, internal career opportunities, job status, and empowerment to improve employee commitment for increasing performance of the forefront employees. All the above variables are predicted in Figure 1.

Based on the research framework, the following research hypothesis of the study was formulated:

**H_1_:** Human capital has a positive relationship with service recovery performance.

**H_2_:** Training has a positive relationship with service recovery performance.

**H_3_:** Rewards have a positive relationship with service recovery performance.

**H_4_:** Teamwork has a positive relationship with service recovery performance.

**H_5_:** Empowerment has a positive relationship with service recovery performance.

**H_6_:** Job description has a positive relationship with service recovery performance.

**H_7_:** Human capital has a positive relationship with employee commitment.

**H_8_:** Training has a positive relationship with service employee commitment.

**H_9_:** Rewards have a positive relationship with service recovery performance.

**H_10_:** Teamwork has a positive relationship with employee commitment.

**H_11_:** Empowerment is related to employee commitment.

**H_12_:** Job description is related to employee commitment.

**H_13_:** Employee commitment has a positive relationship with service recovery performance.

**H_14_:** Employee commitment has a mediating impact between human capital and service recovery performance.

**H_15_:** Employee commitment has a mediating impact between training and service recovery performance.

**H_16_:** Employee commitment has a mediating impact between rewards and service recovery performance.

**H_17_:** Employee commitment has a mediating impact between teamwork and service recovery performance.

**H_18_:** Employee commitment has a mediating impact between empowerment and service recovery performance.

**H_19_:** Employee commitment has a mediating impact between Job description and Service recovery Performance.

### Research Methodology

A quantitative research approach has been applied by using cross-sectional research design. To obtain data, the questionnaires were distributed both directly and online due to the constraints of limited physical interactions during COVID-19 to the respondents by making sure there were no duplicate responses from the same respondents. The takaful insurance industry in Pakistan was surveyed. Respondents answered a series of questions from the questionnaire that had been distributed and the questionnaire was divided into three parts. The first part consisted of demographic profiles (respondent data, roles, background, and political views). The second part consisted of the main independent, mediating, and dependent variables questions which were filled by the respondents. The third part consisted of any suggestions given by the respondents. A total of 500 questionnaires were distributed by using convenient sampling as it is cheap, fast, and easy to get responses (Etikan et al., 2016). Out of these, 350 questionnaires were returned which shows a 70 percent response rate. All of the questions were coded into the excel sheet.

### Research Instrument

The research instrument of the study had been adopted from previous studies where it was already tested. Therefore, this questionnaire had a greater predictive power as compared to a new questionnaire. SRP, the dependent variable, was measured by five items which were adopted from the study of Boshoff and Allen (2000). In addition, the human capital (HC) was measured by six items which were adopted from the study of Mihardjo et al. (2020). Moreover, the Training (TR) was measured by six items which were adopted from the study of Yavas et al. (2003). On the other hand, the rewards (REW) were measured by five items which were adopted from the study of Boshoff and Allen (2000). Teamwork was measured by three items which were adopted from the study of Boshoff and Allen (2000). In addition, empowerment was measured with four items which was adapted from the study of Yavas et al. (2003). On the other hand, job description was measured by four items which were adopted from the study of Mihardjo et al. (2020). All of the variables were measured on a five-point Likert Scale which was ranged from 1 (strongly disagree) to 5 (strongly agree).

### Data Analysis and Interpretation

The inferential analysis of the study was tested by using a Partial Least Square (PLS)-Structural Equation Modeling (SEM). There are various other studies which had also used the PLS-SEM for their analysis (Ahmad et al., 2019c; Siyal et al., 2020, 2021a,2021b). The two models were run for conducting an inferential analysis, measurement model, and structural model.

### Measurement Model

The measurement model of the study could be assessed through convergent and discriminant validity. The convergent validity could be assessed through the factor loadings where the values should be greater than 0.5, the average variance extracted (AVE) value should be greater than 0.5, Cronbach alpha value should be greater than 0.7, and composite reliability value should be greater than 0.7. These values are recommended by various researchers in the previous studies (Hair et al., 2012, 2016). Table 1 predicted values showed that all of the values fulfilled the criteria of recommended values. On the other hand, the discriminant validity could be assessed through the Fornell and Lacker, and Hetrotrait-Monotrait correlation (HTMT). For the assessment of Fornell and Lacker, all the diagonal values should be greater than the values below them. Table 2 predicted values showed that all of the diagonal values are greater than the below values. In addition, for the HTMT the correlation among the construct should be less than 0.85 or 0.90 (Henseler et al., 2015). Table 3 predicted values showed that all of the values are less than 0.85, which shows the discriminant validity of the study.

**TABLE 1:** Measurement model results.

Variable	Item	Loading	Cronbach’s alpha	Composite reliability	AVE
**Service recovery performance**	SRP1	0.838	0.850	0.892	0.675
	SRP2	0.875			
	SRP3	0.817			
	SRP4	0.751			
**Training**	TR1	0.802	0.777	0.855	0.597
	TR2	0.84			
	TR3	0.737			
	TR4	0.703			
	TR5	0.873			
	TR6	0.780			
**Human capital**	HC1	0.731	0.858	0.898	0.639
	HC2	0.781			
	HC3	0.845			
	HC4	0.826			
	HC5	0.809			
**Teamwork**	TW1	0.888	0.834	0.897	0.744
	TW2	0.871			
	TW3	0.827			
**Empowerment**	EMP1	0.823	0.888	0.922	0.748
	EMP2	0.878			
	EMP3	0.884			
	EMP4	0.873	0.847	0.893	0.677
**Job description**	JD1	0.747			
	JD2	0.834			
	JD3	0.879			
	JD4	0.826			
**Rewards**	RE1	0.704	0.890	0.903	0.674
	RE2	0.654			
	RE3	0.759			
	RE4	0.600			
	RE5	0.784			
**Employee commitment**	EC1	0.897	0.904	0.928	O.675
	EC2	0.762			
	EC3	0.567			
	EC4	0.785			
	EC5	0.675			
	EC6	0.871			
	EC7	0.659			
	EC8	0.870			
	EC9	0.631			
	EC10	0.503			
	EC11	0.604			
	EC14	0.804			
	EC16	0.862			
	EC17	0.850			
	EC18	0.764			

*SRP, service recovery performance; TR, training; HC, human capital; RE, rewards; JD, Job description, EMP, empowerment; TW, teamwork; EC, employee commitment.*

**TABLE 2:** Correlation.

	SRP	TR	HC	TW	EMP	JD	RE	EC
**SRP**	0.822							
**TR**	0.176	0.772						
**HC**	0.176	0.468	0.799					
**TW**	0.552	0.14	0.322	0.863				
**EMP**	0.175	0.488	0.624	0.313	0.865			
**JD**	0.004	0.127	0.274	0.036	0.336	0.823		
**RE**	0.512	0.214	0.232	0.163	0.423	0.453	0.752	0.814
**EC**	0.271	0.388	0.424	0.213	0.215	0.341	0.275	0.188

*SRP, service recovery performance; TR, training; HC, human capital; RE, rewards; JD, Job description; EMP, empowerment; TW, teamwork; EC, employee commitment.*

**TABLE 3:** HTMT.

	SRP	TR	HC	TW	EMP	JD	RE	EC
**SRP**								
**TR**	0.126							
**HC**	0.474	0.265						
**TW**	0.453	0.213	0.322					
**EMP**	0.376	0.382	0.644	0.214				
**JD**	0.105	0.323	0.284	0.133	0.135			
**RE**	0.313	0.215	0.222	0.562	0.525	0.153		
**EC**	0.173	0.183	0.324	0.316	0.316	0.441	0.385	

*SRP, service recovery performance; TR, training; HC, human capital; RE, rewards; JD, Job description; EMP, empowerment; TW, teamwork; EC, employee commitment.*

### Structural Model

The purpose of the current study is to test the mediating effect of employee commitment (EC) on the relationship of human research management practices (HRMP) and SRP of the takaful industry in Pakistan. To test this hypothesis, the structural model of the study was assessed by applying the 500-resampling technique. The SEM analysis had shown that human capital (HC) had a positive and significant relationship with the SRP and employee commitment (EC) which supports the proposed hypotheses 1 and 7. In the same vein, training (TR) had also a positive and significant relationship with the SRP and EC, which supports the proposed hypotheses 2 and 8. Similarly, rewards (RE) had also a positive and significant relationship with the SRP and EC that supports proposed hypotheses 3 and 9. Furthermore, teamwork (TW) had also a positive and significant relationship with the SRP and EC, supporting proposed hypotheses 4 and 10. Moreover, the empowerment (EMP) had also a positive and significant relationship with the SRP and EC that supported hypotheses 5 and 11. Again, Job description (JD) also had a positive and significant relationship with the SRP and EC and so supported hypotheses 6 and 12. Lastly, EC also had a positive and significant relationship with the SRP, supporting hypothesis 13. These above-discussed findings show that human resource management practices (HRMP) are playing an important role to increase the SRP of the tankful industry in Pakistan. These findings are in line with the following previous studies where HRMP had a significant relationship with the SRP (Mihardjo et al., 2020).

**FIGURE 2:**
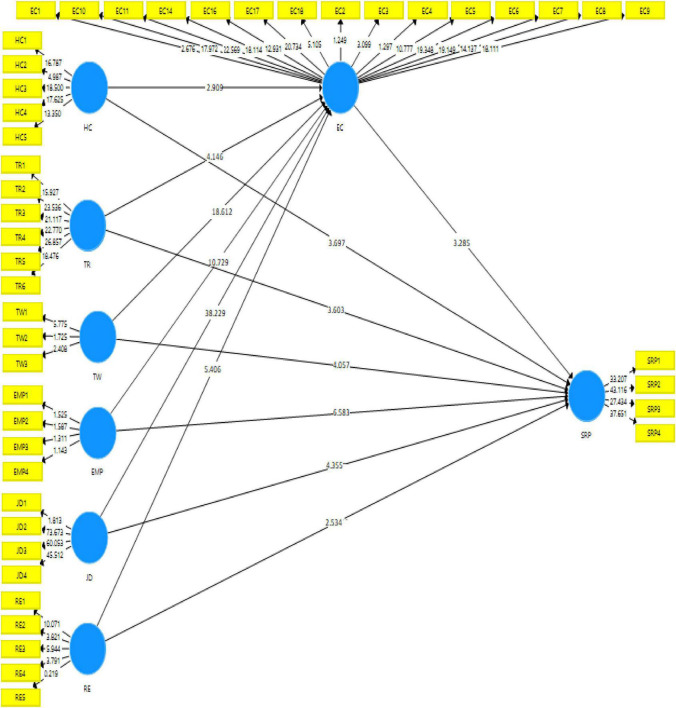
Structural Model.

In other words, the indirect effect had shown that EC had a partial mediating effect on the relationship of HC, TR, RE, TW, JD, and SRP while having an insignificant relationship between SP and SRP. These findings had shown that EC is considered to be a significant mediating effect on the relationship of HRMP and SRP. These findings had shown that HRMP are considered to be a more important component to increase takaful insurance SRP at the time of the COVID-19 pandemic. However, WE was not significantly mediating on the relationship between EMP and SRP. A possible reason for this explanation is that there could be an overlapping of other variables on the relationship of EMP and SRP because the EMP had a direct significant and positive effect on SRP. Another, possible reason for this explanation is that respondents did not give importance to the EC as a mediating variable on the relationship of EMP and SRP. Therefore, these findings have shown that there is a need for another variable between their relationships. Based on these findings, the EC is considered to be a significant mediating variable because it has a significant mediating effect on most of the exogenous and endogenous variables. These findings are further in line with the previous studies where EC had a significant mediating effect on the relationship of exogenous and endogenous variables (Mihardjo et al., 2020). All above-discussed values are predicted in Table 4 and Figure 2.

**TABLE 4:** Direct and indirect effect results.

Hypotheses	Path	Beta	STDEV	T statistics	*P*-values
**H1**	**HC−> SRP**	0.146	0.04	3.697	0.000
**H2**	**TR−> SRP**	0.180	0.050	3.603	0.000
**H3**	**RE−> SRP**	0.098	0.039	2.534	0.012
**H4**	**TW−> SRP**	0.220	0.054	4.057	0.000
**H5**	**EMP− > SRP**	0.329	0.050	6.583	0.000
**H6**	**JD− > SRP**	0.162	0.037	4.355	0.000
**H7**	**HC−> EC**	0.16	0.055	2.909	0.004
**H8**	**TR−> EC**	0.151	0.036	4.146	0.000
**H9**	**RE−> EC**	0.293	0.054	5.406	0.000
**H10**	**TW−> EC**	0.372	0.02	18.612	0.000
**H11**	**EMP− > EC**	0.285	0.027	10.729	0.000
**H12**	**JD− > EC**	0.602	0.016	38.229	0.000
**H13**	**EC− > SRP**	0.234	0.490	3.285	0.000
**H14**	**HC−> EC- > SRP**	0.117	0.015	7.603	0.000
**H15**	**TR−> EC- > SRP**	0.705	0.024	28.945	0.000
**H16**	**RE-> EC- > SRP**	0.108	0.013	8.021	0.000
**H17**	**TW− > EC- > SRP**	0.400	0.041	9.816	0.000
**H18**	**EMP− > EC- > SRP**	0.044	0.088	0.506	0.613
**H19**	**JD− > EC- > SRP**	0.248	0.059	4.181	0.000

*SRP, service recovery performance; TR, training; HC, human capital; RE, rewards; JD, Job description; EMP, empowerment; TW, teamwork; EC, employee commitment.*

## Discussion and Conclusion

This study was intended to investigate the mediating effect of employee commitment (EC) on the relationship of human resource management practices (HRMP) and SRP of the takaful insurance industry in Pakistan during the COVID-19 pandemic. The results of this study revealed that HRMP such as (human capital, employee training, rewards, empowerment, job description, and teamwork) significantly influence the SRP, particularly with the mediating role of the SRP during the pandemic. The results of this study are supported by previous studies (Piaralal et al., 2014; van Esch et al., 2018; Mihardjo et al., 2020) conducted to investigate the SRP toward service performance. The research is focused on three distinct fields of literature. First, the investigation finds positive links in the takaful industry in Pakistan between HRMP. The results of the study encourage the managers to introduce the general and potentially relevant practices into the insurance community of Pakistan. Secondly, this study shows that the creative approach has an increased effect on a few HRMPs that boost SRP, consistent with the earlier studies on strategic management of human resources (van Esch et al., 2018).

### Implications

From a theoretical standpoint, Pakistani researchers can use this model in future research in service sector fields such as banking, retailing, and hotels to replicate and compare this finding. Currently, there is limited research which is available on the relationship of HRMP, EC, and SRP and this research indicates that more research is needed. The model needs to be improved further in order for employees to understand the requirements for SRP. Furthermore, the instruments used in this study are valid and reliable. The majority of the findings in this study are similar to those found in plenty of other studies. Moreover, the study contributes to strategic HRMP literature in Pakistan, especially in the takaful insurance industry. Moreover, in the relationship between the performance of the market in the business, the EC also has some serious effects. The present conditions in Pakistan’s takaful industry showed undeniable and stable results. Owing to this, most of the Pakistani takaful insurance companies are in their very early stages and the management structure is in an incubatory phase. In addition, organizations need to do what they can to incorporate high human resource management practices and genuinely combine these activities into their core strategies to lead to creative strategies. As the results of this study show, this should improve firm efficiency. According to Schlegelmilch et al. (2003), management focuses on ensuring that human resource management practices in the external environment are optimally integrated and that the organization is prevented from any significant damage to the violent change method. Considering the setting of Pakistan-based takaful insurance companies, the results of this study highlight the external fit of human resource practices which can boost company efficiency and contribute to creative business strategy.

Moreover, this research also suggests a number of important managerial or practical implications. HRMP (Human capital, rewards, training, teamwork, job description, and empowerment) are significant predictors of SRP by takaful insurance customer service employees. EC was also discovered to be a mediator, influencing the relationship between HRMP and SRP. As a result, management in the takaful insurance industry should clearly design and implement a variety of organizational strategies. Managers should establish high standards for superiority in service delivery confidence in customer service employees for making decisions to increase the level of SRP. As a result, management should take steps to focus more on HRMP that can help increase EC, which will increase SRP and will recover service failures. Furthermore, it will allow managers to spend less time dealing with customer complaints and more time focusing on other policy issues (Babakus et al., 2003).

### Limitation and Future Research

The study also has some limitations. The research is firstly limited to the takaful insurance industry during the COVID-19 pandemic but the factors effect on the conventional insurance industry were not explained. Therefore, the generalization of results to other industries and in a more normal situation in Pakistan is therefore limited. In this regard, a comparative study between the Islamic and conventional insurance industries could be interesting in upcoming research. Research can be done in the future with the aid of other Pakistani industries under normal conditions. Secondly, this analysis is based on a cross-sectional design and the results are not enough to say that all well-performing organizations in Pakistan currently are using HRMP. Longitudinal studies should also be undertaken in the future to support the point that human resource management practices affect organizational output directly. Thirdly, different facets of organizational success may be considered in future research to explore the impacts of HRMP on them. Fourthly, the study was limited on mediating effect, there are several other variables like organization culture that could moderate and meditate the relationship of HRMP and SRP. Therefore, future research could consider some other moderating variables in the correlation between HRMP and SRP. Among these relationships, the research recommends employee gender and personality traits as moderating variables that could increase the moderator predictive power. Finally, from a methodological perspective, a convenience sampling technique was being used in this research which consisted of 350 responses, but issues like the location and representativeness of the sample might effect the findings. Consequently, it would be useful in upcoming research to overcome the current research limitations by attaining a comprehensive sample of customer service employees and a probability random sampling technique that could help to give more comprehensive results.

## Conclusion

Numerous studies had been done regarding HRMP in developed economies, namely in the areas of financial, loadings, and retail, but there was limited literature especially in the takaful insurance industry. Moreover, the results also have an inconsistent finding and failed to obtain conclusive results that could be generalized in the Pakistan context. Therefore, we have done our research to investigate the factors influencing the SRP of the takaful insurance industry setting from the perspective of HRMP. This study also investigates the mediating effect of EC as a mediator on the relationship between HRMP and SRP. By doing so, the study made dynamic and clearly articulated contributions because most of the direct and indirect hypotheses of the study are supported. Therefore, the findings of this study could increase and extend our acceptance of variables that affect SRP. Continued study is needed to improve this research and address the limitations of current research. As a result, it is anticipated that this research will provide preliminary insight and understanding on the factors influencing SRP in the Pakistani takaful insurance industry.

## Data Availability Statement

The raw data supporting the conclusions of this article will be made available by the authors, without undue reservation.

## Ethics Statement

Ethical review and approval was not required for the study on human participants in accordance with the local legislation and institutional requirements. Written informed consent from the patients/participants OR patients/participants legal guardian/next of kin was not required to participate in this study in accordance with the national legislation and the institutional requirements.

## Author Contributions

SS and JM designed and conducted the whole research. SS did write-up and analysis. RA did analysis. SQ did editing. MJ and CX scientific revisions. JM secured funding to conduct this research. All authors contributed to the article and approved the submitted version.

## Conflict of Interest

The authors declare that the research was conducted in the absence of any commercial or financial relationships that could be construed as a potential conflict of interest.

## Publisher’s Note

All claims expressed in this article are solely those of the authors and do not necessarily represent those of their affiliated organizations, or those of the publisher, the editors and the reviewers. Any product that may be evaluated in this article, or claim that may be made by its manufacturer, is not guaranteed or endorsed by the publisher.
